# Non-Immunologic Causes of Late Death-Censored Kidney Graft Failure: A Personalized Approach

**DOI:** 10.3390/jpm12081271

**Published:** 2022-08-01

**Authors:** Claudio Ponticelli, Franco Citterio

**Affiliations:** 1 Independent Researcher, Via Ampere 126, 20131 Milano, Italy; ponticelli.claudio@gmail.com; 2 Renal Transplant Unit, Fondazione Policlinico Universitario A. Gemelli, IRCCS, Università Cattolica Sacro Cuore, 00168 Roma, Italy

**Keywords:** hyperuricemia, quality of the donated kidney, adherence to prescriptions, drug toxicities, arterial hypertension, dyslipidemia, new onset diabetes mellitus, hyperu

## Abstract

Despite continuous advances in surgical and immunosuppressive protocols, the long-term survival of transplanted kidneys is still far from being satisfactory. Antibody-mediated rejection, recurrent autoimmune diseases, and death with functioning graft are the most frequent causes of late-kidney allograft failure. However, in addition to these complications, a number of other non-immunologic events may impair the function of transplanted kidneys and directly or indirectly lead to their failure. In this narrative review, we will list and discuss the most important nonimmune causes of late death-censored kidney graft failure, including quality of the donated kidney, adherence to prescriptions, drug toxicities, arterial hypertension, dyslipidemia, new onset diabetes mellitus, hyperuricemia, and lifestyle of the renal transplant recipient. For each of these risk factors, we will report the etiopathogenesis and the potential consequences on graft function, keeping in mind that in many cases, two or more risk factors may negatively interact together.

## 1. Introduction

Today, kidney transplantation is the preferred treatment for patients with end-stage kidney disease. Declining rates of acute rejection have led to improvements in short-term kidney transplant survival, but long-term results are still far from being satisfactory. Apart from death with functioning graft, chronic antibody-mediated rejection and recurrence of autoimmune diseases are the major limits for long-term graft survival [1,2,3,4,5]. For this reason, physicians’ attention in the follow-up of transplant recipients is generally focused on immune factors, whereas nonimmunologic factors receive less attention, despite their substantially high contribution to post-transplant morbidity.

In this paper, we will review the most important nonimmunologic causes of death-censored kidney allograft failure.

### 1.1. Quality of the Donated Kidney

Renal function in renal transplant recipients (RTRs) in general is supported by a solitary functioning kidney. In the case of a deceased donation, the estimated glomerular filtration (eGFR) of the donor usually ranges between 60 and 100 mL/min/1.73 m^2^, but in older and extended criteria donors (ECDs) the eGFR may be considerably lower. Although any functioning kidney transplant is cost-effective, compared to remaining on dialysis on a waitlist [6], there is evidence that subclinical functional defects are generated in a solitary kidney leading to long-term progressive vasculo-glomerular and tubulo-interstitial lesions, also in nontransplanted individuals [7,8]. Early after transplantation, brain death cytokine storm, ischemia-reperfusion injury, acute rejection, and infections [9,10,11,12,13,14] may lead to acute kidney injury (AKI), ending in a chronic or acute inflammatory state concurring in the pathogenesis of arterial hypertension [15,16]. Another major consequence of the inflammatory state is the development of chronic hypoxia that through the mediation of interleukine 1 and 6, angiotensin II, and transforming growth factor beta can result in excessive accumulation of extracellular matrix and fibrosis [17,18,19]. The excessive deposit of the extracellular matrix can induce epithelial-mesenchymal transition and lead to interstitial fibrosis and chronic allograft dysfunction [20,21,22].

These complications are more frequent and severe in transplants from deceased donors, particularly ECDs. Extended criteria donors nowadays are a main source for kidney transplantation. Survival and quality of life are better for ECD kidney transplants in comparison with dialysis at any age [23,24,25]. However, the number of nephron and GFR progressively decreases after the age of 29 [26], favoring the development of hypertension and CKD in the general population. In kidney transplantation, large series reported that a higher age of donor is related to a less kidney survival at 5 years [27]. For this reason, organs from elderly donors are mainly allocated to elderly recipients, unless the older donor has no history of hypertension, no increased creatinine, no cerebrovascular death, no diabetes or cancer, and no other reasons to be defined as a marginal donor [28].

Living donor transplants in general are followed by a lower release of pro-inflammatory factors in comparison with a deceased donor transplant. Cold ischemia time, ischemia-reperfusion injury, cytokine and autonomic storms related to brain death, are considerably reduced in living donor transplants. Preemptive transplantation also reduces the proinflammatory status, mainly linked to dialysis [29,30].

### 1.2. Adherence to Prescriptions

Medication non-adherence is prevalent in around 39% of patients with CKD and is associated with poor control of blood pressure, disease progression, adverse events, and mortality [31]. In RTRs the major problem is the complexity of treatment even in patients with stable graft function. The burden of pills that patients receive is not only due to immunosuppressive regimens but also to hypertension, dyslipidemia, and anemia. In a survey of 1130 RTRs with stable serum creatinine, an average 5.9 years after transplantation, each patient was taking on average 11 pills per day, 6 immunosuppressant and 5 non-immunosuppressant [32]. Many immunosuppressive agents used in organ transplantation exert adverse side effects including gastrointestinal disorders, hyperglycemia, hypertension, and possible neurologic complications. Another issue that transplant physicians should be aware of is depression, which is present in 25% of RTRs [33,34]. The presence of depressive symptoms negatively affects adherence to prescriptions and clinical outcomes. Poor adherence is particularly frequent in adolescents, with a weighted prevalence of non-adherence in 32% of RTRs younger than 21 years [35]. In adult RTRs non-adherence to immunosuppressive medications is common, with 20% to 55% of patients being non-adherent [36,37,38]. Poor adherence is often related to social isolation, low belief in medications and/or poor socio-economic conditions. Unintentional compliance is frequent in older patients. They often forget to take medications, and the greater the number of drugs to take, the higher the risk of forgetfulness. Moreover, changes in the dosage or type of medications can often be misunderstood. A systematic review showed that nonadherence was associated with poor clinical outcomes, contributing to 20% of late rejection episodes and 16% of graft losses [39]. Poor adherence to clinical visits and/or to nonimmunosuppressive medications may favor the occurrence of infection, tumors, and CVD. Noncompliance is also favored by the complexity of the immunosuppressive regimen prescribed. Many immunosuppressive drugs are prescribed twice a day, irrespective of available once-a-day formulations and of their pharmacokinetic profile. Studies on patients receiving antibiotics have clearly shown that compliance is strictly related to prescriptions schedules. Morning once-a-day administrations had the best patient compliance. A meta-analysis of randomized controlled trials showed that compliance to antibiotic treatment is higher when an antibiotic is administered once a day than multiple times daily for the treatment of specific infections and for specific classes of antibiotics [40]. Lifetime treatment with immunosuppressive drugs should be personalized and designed, keeping in mind simplicity, to enhance patient compliance.

### 1.3. Drug Toxicities

**Figure 1:**
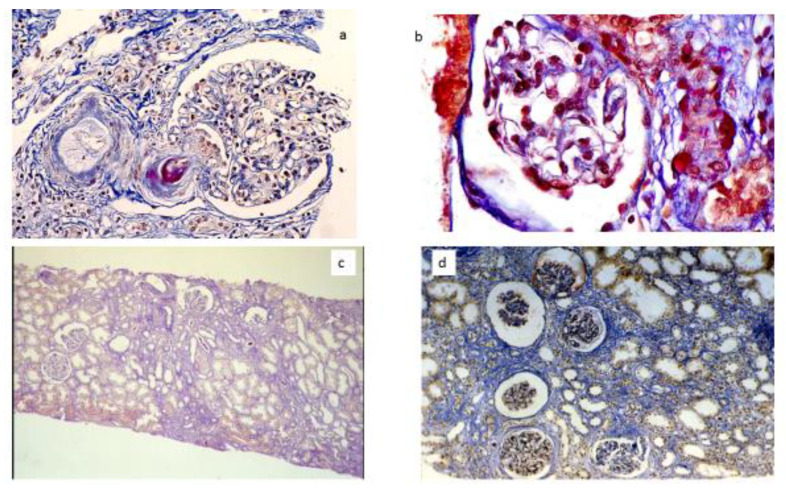
CNI renal toxicity. (**a**) CNI arteriolopathy; a preglomerular arteriole showing mucinoid thickening of the arteriolar wall. (**b**) CNI arteriolopathy with severe nodular hyalinosis of the wall. (**c**) Striped interstitial fibrosis, tubular dilatation and atrophy. (**d**) Diffuse interstitial fibrosis, glomerular ischemia and sclerosis.

Calcineurin inhibitors (CNI) may be responsible for progressive and irreversible kidney toxicities. CNI can cause kidney vasoconstriction by increased expression of vasoconstrictor factors, such as angiotensin II, endothelin-1, thromboxane A2, and leukotrienes, while reducing the production of vasodilators, such as prostacyclin, prostaglandin E2, and nitric oxide. In the long term, a chronic nephrotoxicity may develop, characterized by interstitial fibrosis, tubular atrophy, glomerular sclerosis, and afferent arteriolopathy (Figure 1) [41]. Nephrotoxicity is usually dose-dependent, related to intra- and inter-individual bioavailability and sensitivity. In the 1980s, during first experiences, cyclosporine (CsA) in kidney transplantation was used at elevated doses, up to 15 mg/kg/day. High dosages resulted in frequent acute and chronic nephrotoxicities. An Australian study pointed out that 58% transplanted kidneys treated with CsA for 10 years showed histologic signs of dose-dependent chronic toxicities, such as arteriolar lesions, patchy interstitial fibrosis, tubular atrophy, and focal, segmental, and global glomerular sclerosis [42]. In the same way, when tacrolimus (TAC) was introduced as an immunosuppressive agent, target levels were in the range of 15–20 ng/mL. Slowly, CSA and TAC dosage and blood target levels were considerably reduced. Today, initial doses of CsA and TAC do not exceed 3–5 mg/kg/day and 0.1 mg/kg/day, respectively. Doses are then progressively reduced over the time, up to minimal effective doses for maintenance. To prevent nephrotoxicity, it is also important to avoid concomitant use of nephrotoxic drugs (quinolones, aminoglycosides, amphotericin B, foscarnet, etc.) or drugs that modify the pharmacokinetics of CNI, interfering with cytochrome P 450 activities. The role of CNI as a main cause of interstitial fibrosis has been overevaluated as many authors have suggested [1,43,44]. High CNI exposure can also lead to systemic effects, including arterial hypertension, dyslipidemia, glucose intolerance, hyperuricemia, indirectly affecting kidney allograft function.

An important, still unsolved issue in CNI monitoring is the relationship between current therapeutic drug monitoring based on blood levels and the intracellular concentration of CNI. CNI exert their action inside T lymphocytes. Studies show that blood levels do not reflect the intracellular concentration of CNI and may be misleading [45]. In addition, one should take into account the impact of concurrent drugs that can interfere with CYP450 or P glycoprotein, thus modifying the blood levels [46]. An expert consensus pointed out that acute rejection and kidney toxicity still occur in patients showing blood CNI concentrations within the therapeutic range [47]. Because CNIs exert their action inside T lymphocytes, intracellular CNIs should provide reliable results. However, measuring intracellular CNIs concentrations is not easy. To date, there are no guidelines for the TDM of intracellular CNI concentrations. A recent clinical study in kidney transplantation looked at the relationship between intracellular TAC concentration and rejection or drug-related toxicities. Tacrolimus was measured in the blood and within the cells on days 3 and 10 after kidney transplantation, and on the morning of a for-cause kidney transplant biopsy. The correlation between TAC in the cells and TAC in the blood was poor. The study conclusion was that TAC concentration in cells was not significantly associated with the occurrence of rejection. These results might be inconclusive because of the low number of patients included in this study and because peripheral blood mononuclear cells are not a specific enough matrix to monitor tacrolimus [48]. In the future, the combination of pharmacokinetics, pharmacogenetics, pharmacodynamics, and immunologic biomarkers may allow a better guide the use of TAC in RTRs. The inhibitors of the mammalian target of rapamycine (mTOR), sirolimus, and everolimus can exert dose-dependent antiproliferative and apoptotic effects on epithelial tubular cells [49]. Impaired tubular reabsorption of albumin has been demonstrated in a transplant patient treated with sirolimus in 2006 [50]. A randomized controlled trial comparing CsA with sirolimus reported increased proteinuria, increased urinary excretion of markers of tubular damage and evidence of tubular injury on kidney biopsy in patients treated with sirolimus [51]. Taken together, these data would speak in favor of a tubular toxicity leading to poor reabsorption of albumin and small proteins. Other immunosuppressive drugs used in kidney transplantation do not exert direct nephrotoxicity, including belatacept, purine synthesis inhibitors, proteasome inhibitors, and polyclonal and monoclonal antibodies.

### 1.4. Dialysis Vintage

*End-stage renal disease (ESRD) is a wasting illness and is often associated with a higher risk of cardiovascular diseases and many other comorbidities.* A retrospective analysis of the United States Renal Data System Registry reported that compared to preemptive transplantation, waiting times of 0 to 6 months, 6 to 12 months, 12 to 24 months and over 24 months conferred a 17%, 37%, 55%, and 68% increase in risk, respectively, for death-censored graft loss after transplantation [52]. Poor graft function risk is particularly increased in elderly patients [53].

### 1.5. Hypertension

**Figure 2:**
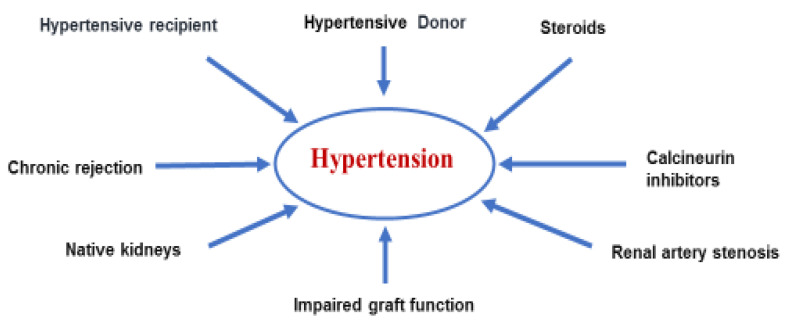
Main causes of post-transplant hypertension.

Arterial hypertension is frequently observed in RTRs and pathogenesis is multifactorial in most cases (Figure 2). Many patients are already hypertensive before transplantation and CNI immunosuppression may induce de novo or worsen previous hypertension. CNI, as a side effect, activates the renin–angiotensin system and deactivates the atrial natriuretic peptide, leading to arteriolar vasoconstriction with consequentially reduced GFR and extracellular fluid expansion. [54]. Additional causes of hypertension are steroid-induced water and salt retention, further aggravated by increased extracellular volume and renin production caused by impaired graft function. Other causes may also contribute to the development of post-transplant hypertension: native kidney disease, old donor age, chronic rejection, and transplant renal artery stenosis. All these factors contribute to extracellular expansion and increase in cardiac output. In the meantime, there is an increase in peripheral vascular resistance caused by inappropriate secretion of renin-angiotensin-aldosterone axis [55].

Arterial hypertension not only increases the risk for cardiovascular events but can also deteriorate kidney allograft function. Several studies have shown that the higher the levels of blood pressure are, the higher is the risk of graft failure [56,57,58,59,60]. Kasiske et al. [61] found that a 10-mmHg increment above 140 mmHg in systolic blood pressure was associated with a 12% relative risk for graft failure and 18% relative risk of death. On the other hand, good blood pressure control may prevent many cardiovascular and kidney complications. Appropriate lifestyle behaviour and physical activity is the first step to control hypertension. Diuretics exert anti-hypertensive effect by reducing salt and water overload but in transplant recipients this may cause a drop in GFR due to the impaired hemodynamic adaptation of the transplanted kidney. Calcium channel blockers reduce systemic vascular resistance acting on vascular smooth cells and may protect one from CNI-induced vasoconstriction. Renin–angiotensin system (RAS) inhibitors control arterial hypertension, reduce proteinuria, and may treat erythrocytosis, but a meta-analysis of three randomized controlled clinical trials and two cohort studies, including 20.024 RTRs, showed no significant reduced risk of allograft loss or mortality in RTRs treated with RAS inhibitors [62]. Current convincing data to prove that RAS inhibitors can actually improve outcomes in RTRs are still lacking. Some transplant physicians are reluctant to prescribe these agents, as potassium can be increased, and hemoglobin and GFR can be reduced. To obtain good control of hypertension and to avoid side effects, most kidney transplant recipients are receiving a synergistic combination of antihypertensive drugs.

### 1.6. Dyslipidemia

Dyslipidemia is a common complication after kidney transplantation [63,64] and is an important contributor to the high rate of cardiovascular diseases in RTRs, also potentially causing kidney dysfunction. Typically, dislipidemia is observed during the first 3 to 6 months after transplantation, when freedom from dialysis allow a free diet, and may then persist for 10 or more years. The pathogenetic mechanisms for lipid disorders after transplantation are multifactorial: excessive dietary intake of saturated fat and eating disorders, poor physical activity, and sedentary habits are common in RTRs. Many transplant recipients become overweight or obese after transplantation [65] and develop diabetes after transplantation [66]. Moreover, poor post-transplant renal function, leading to CKD stage 3 [67], can favor the development of dyslipidemia [68]. However, the most important cause of post-transplant dyslipidemia is due to immunosuppressive drugs: CSA, TAC, and mTOR inhibitors. Cyclosporine increases total cholesterol (C), VLDL-C, and LDL-C by downregulating LDL receptor expression [69]. Hypertriglyceridemia in CNI-treated RTRs is associated with increased plasma apoCIII concentrations [70]. Despite hypercholesterolemia being significantly less frequent in RTRs receiving TAC with respect to CsA [71], tacrolimus can significantly increase plasma triglycerides, reducing LPL activity [72]. Moreover, mTOR inhibitors are frequently associated with dyslipidemia [73]. These drugs increase hepatic synthesis of apoB100, VLDL, expression of adipose tissue lipase, apoCIII, and lipophagy, while decreasing LDL liver catabolism, LPL expression, and preventing the uptake of lipids into adipocytes [74,75,76]. On the other hand, dyslipidemia caused by mTOR inhibitors may be balanced by the cardioprotective effects of these drugs [67]. Many transplant recipients receive lifetime maintenance glucocorticoids that enhance the activity of acetyl coenzyme convertase and of fatty acid system, increasing hepatic synthesis of VLDL, downregulating LDL receptor activity, and inhibiting lipoprotein lipase [77]. Very few transplant recipients are on a steroid-free regimen. Evidence suggesting that renal lipid accumulation and lipotoxicity may lead to kidney dysfunction has mounted significantly in recent years. Lipid accumulation, changes in circulating adipokines, alterations in renal lipid metabolism, insulin resistance, generation of reactive oxygen species, and endoplasmic reticulum stress are factors eventually leading to damage of the glomerular filtration barrier and to kidney failure [78,79]. Other studies showed that an excessive accumulation of cholesterol and/or triglycerides may cause podocyte injury and proteinuria, suggesting that lipids represent a major regulator of danger signaling from the circulation to glomerular cell [80]. In a retrospective study conducted in more than 12,000 healthy participants, high triglycerides and low HDL-C predicted an increased risk of kidney dysfunction, and treatment of these abnormalities could reduce the incidence of early renal disease [81]. Finally, decreased fatty acid oxidation in CKD may contribute to lipid accumulation in the tubular compartment, which results in energy depletion, followed by apoptosis and de-differentiation, all factors contributing to fibrosis and CKD progression [82]. Although it is difficult to extrapolate these data to RTRs, few studies reported associations between early post-kidney transplant lipid levels and subsequent damages of graft function or death-censored graft loss [83,84]. Pharmacological treatment is necessary if LDL cholesterol level is > 190 mg/dL. Statins are generally well tolerated but myositis and muscle symptoms may occur, leading to poor adherence or discontinuation. There is evidence that statins may reduce cardiovascular events, whereas the benefits on kidney function are controversial. A meta-analysis of randomized controlled trials reported that in adults with CKD, statins do not reduce the risk for kidney failure events, but may modestly reduce proteinuria and rate of eGFR decline [85,86]. Drug–drug interactions that increase statin plasma concentrations mainly involve the co-administration of inhibitors of cytochrome P enzymes (particularly CYP3A4, which is inhibited by CNI), or inhibitors of the transporter proteins activities, which participate in statin cell influx and efflux. Although CNI may increase blood levels of lipophylic statins, with the exception of fluvastatin, results from several studies show that statins do not induce increased systemic exposure of CNI. Ezetimibe represents an alternative, especially for statin-intolerant patients or when added to the highest tolerated statin dose. If a predominant hypertriglyceridemia is present, low calorie intake, low-fat diet and fish oil is suggested and may be beneficial. Fibrates may also lower triglyceride levels but can be responsible for a reversible increase in serum creatinine. There is insufficient information on the use of monoclonal antibodies in RTRs [87].

### 1.7. New Onset Diabetes Mellitus

New onset diabetes after transplantation (NODAT) is a frequent complication in RTRs, due by traditional and non-traditional pro-diabetic risk factors. The traditional risk factors are the same leading to development of diabetes type 2 in the general population. Non-traditional risk factors include perioperative stress, hepatitis C infection, cytomegalovirus infection, vitamin D deficiency, hypomagnesemia, and immunosuppressive medications, such as glucocorticoids, CNI, and mTOR inhibitors. Glucocorticoids may induce insulin resistance by different mechanisms, including impaired osteoblast function with consequent increase in visceral adiposity and lipolysis, leading to elevated free fatty acids [88], hepatic steatosis [89], and decreased transcription of insulin receptors in skeletal muscle, while increasing transcription of two proteins that counter insulin action [90]. CNI can induce decreased insulin secretion [91], increased insulin resistance [92], and direct toxicity on β cells [93]. The diabetogenic effects of TAC are more intense compared with those of CsA [94]. Immunosuppression with mTOR inhibitors has a direct effect on pancreatic beta cells, reducing directly insulin secretion [95], and inducing gluconeogenic pathways in the liver [96]. However, in RTRs the association between mTOR inhibitors medication and hyperglycemia is weak, and is mostly due to the contemporary drug interaction with CNI [97]. NODAT may progress to diabetic nephropathy and proteinuria. The kidney pathological findings of NODAT are similar to those of primary diabetic nephropathy in native kidneys [98]. However, NODAT is frequently associated with vascular or tubulointerstitial histological changes due to concurrent rejection, viral infections, or drug nephrotoxicity. Avoiding excessive weight gain, leading a healthy lifestyle, reducing caloric intake, and engaging in physical exercise are typically recommended at the discharge of patients after renal transplantation, but compliance with these indications is generally low. Management of diabetes is more challenging in transplant recipients than in nontransplant patients, because of the greater risk on kidney function. Metformin may offer some advantages over other glucose-lowering agents with respect to risk of hypoglycemia [99] and beneficial effects on the kidney [100,101], There has been concern about the risk of life-threatening lactic acidosis. However, many kidney transplant recipients are using metformin without experiencing side effects [102]. Two comprehensive reviews found no evidence of an increased risk of lactic acidosis, using metformin compared to other anti-hyperglycemic treatments [103,104]. Potential side effects of rosiglitazone and pioglitazone include edema, congestive heart failure, and bone fractures [105,106]. Glucagon-like peptide-1 (GLP-1) inhibitors may reduce progression of renal disease in type 2 diabetes [107,108], but these drugs have serious gastrointestinal effects and might increase the risk of pancreatitis and/or tumors [109]. A small study in organ transplant recipients reported that GLP-1 inhibitors are effective and do not affect TAC levels or transplant outcomes in the short term [110]. Inhibitors of dipeptidyl peptidase 4 (DPP-4 inhibitors or gliptins) are generally considered safe, with a low rate of side effects. Concerns about increased risk of cardiovascular events or pancreatic cancer development have not been confirmed [111]. A systematic review of 7 studies reported that DPP-4 inhibitor use in transplant recipients did not result in significant change in eGFR or TAC blood levels [112]. Sodium glucose cotransporter-2 (SGLT2) inhibitors, such as canagliflozin, dapagliflozin, empaglifloziin, sotagliflozin etc., inhibit the reabsorption of glucose in the proximal tubular cells and facilitate glucose excretion in urine. As glucose is excreted, its plasma levels fall, leading to an improvement in all glycemic parameter. SGLT2 inhibition has also been shown to reduce cardiovascular mortality and preserve kidney function in patients with type 2 diabetes [113]. However, urinary tract infections and a slight initial decrease in renal function may limit use of SGLT2 inhibitors [114]. A systematic review and meta-analysis of 8 studies with 132 RTRs with excellent kidney function reported that SGLT-2 inhibitors for treatment of NODAT are effective in lowering glycate hemoglobin, reducing body weight, and preserving kidney function without serious adverse events [115]. In RTRs requiring insulin treatment, the dose and type of insulin prescription should be based on individual patient needs.

### 1.8. Hyperuricemia

Hyperuricemia is related to cardiovascular diseases [116,117] and is an independent predictor of chronic kidney disease development and progression [118,119,120,121,122]. An independent association between serum urate levels and allograft outcomes was found in RTRs [123,124]. A retrospective analysis in 2993 kidney transplant recipients showed that low and normal serum urate levels within the first year are an independent predictor of better renal allograft outcomes in the long term [125]. However, in RTRs it is difficult to separate hyperuricemia as a cause or consequence of kidney injuries [126]. The mechanistic interpretation of urate-related kidney injury is complex. Hyperuricemia can activate the renin-angiotensin-aldosterone system, along with the inhibition of nitric-oxide synthesis in the kidney, leading to medial thickening of preglomerular arterioles, renal vasoconstriction, and increased systemic blood pressure [127]. On the other hand, uric acid is recognized as an endogenous damage-associated molecular pattern by pattern-recognition receptors with engagement of inflammasomes and activation of pro-inflammatory interleukin-1β and interleukin-18 [128]. This inflammatory process may end in kidney ischemia and hypoxia, two powerful inducers of tubulointerstitial fibrosis [129], and in the epithelial-mesenchymal transition of renal tubular cells with increased fibronectin synthesis [130,131]. In summary, a two-hit model can be proposed. The first hit entails activation of molecules and factors that promote endothelial dysfunction, proliferation of vascular smooth-muscle cells and sodium reabsorption, leading to increased systemic blood pressure. The second hit involves activation of inflammatory status increasing vascular resistances, kidney ischemia and hypoxia, eventually leading to vascular and tubulo-interstitial lesions inducing development and progression of kidney disease [132]. Xanthine oxidase inhibitors are the cornerstone to reduce uricemia to ≤6 mg/dL, the target value for urate-lowering drugs. Allopurinol is a nonspecific competitive inhibitor of xanthine oxidase that undergoes conversion to oxypurinol, prior to renal excretion. Serious adverse events are mainly related to hypersensitivity to allopurinol [133]. Concomitant administration of azathioprine and xanthine oxidase inhibitors engenders the hazard of severe bone marrow suppression because xanthine oxidase inhibitors inhibit the oxidation of 6-mercaptopurine to inactive metabolites. Therefore, concomitant administration should be avoided, unless allopurinol is the only option and has to be used. In these cases, dose reduction of azathioprine or 6-mercaptopurine is suggested, and white blood cells should be strictly monitored. More recently, febuxostat has become an established alternative for the treatment of hyperuricemia. Febuxostat is extensively metabolized by oxidation and acyl-glucuronidation, with subsequent renal clearance of febuxostat-acyl-glucuronides. Although pharmacokinetic parameters are not affected by mild to moderate hepatic impairment, there is no consensus on whether renal impairment has any effect on the pharmacokinetics of febuxostat [134]. As for allopurinol, current febuxostat labeling contraindicates the concomitant administration of febuxostat with either azathioprine or 6-mercaptopurine. In RTR, febuxostat had higher odds to reach the target of serum uric acid < 6 mg/dL compared to allopurinol, without causing significant side effects [135,136].

Although mounting evidence indicate that hyperuricemia may concur with other factors in inducing kidney damages, no robust data are available to support the routine use of pharmacotherapy for RTRs with asymptomatic hyperuricemia.

### 1.9. Anemia

In transplant recipients with poor kidney allograft function, anemia is frequent. Erythropoiesis stimulating agents (ESAs) may be used in the early posttransplant period to prevent renal hypoxia or in the late period to prevent cardiovascular complications. There is no evidence that treatment with ESAs may affect kidney function in RTRs. Rather, targeting higher hemoglobin levels may reduce the progression of allograft nephropathy [137].

## 2. Lifestyle

### 2.1. Smoking

Epidemiological studies documented a marked risk of irreversible proteinuria in smokers [137,138]. Smoking can also accelerate the progression of renal failure in patients with kidney disease [139]. Patients who continue to smoke after transplantation are at increased risk of graft failure [140] that may be reversed by stopping smoking [141]. A history of smoking before kidney transplantation can also contribute significantly to allograft loss [142,143]. The kidney histologic picture in smokers is characterized by interstitial fibrosis [144] or nodular glomerulosclerosis [145]. The pathogenesis of smoking-related renal damage is largely unknown. The intermittent increase in blood pressure during smoking might play a major role in causing renal damage [146]. Increased sympathetic activity, increased renal vascular resistance, oxidative stress, increased intraglomerular pressure, and/or renal artery arteriosclerosis probably contributes to the deleterious effects of smoking [147].

### 2.2. Sedentary Activity

A retrospective study evaluated the impact on kidney function of active physical activity (≥ 30-min, 5 times/week) versus nonactive patients in 2060 stable RTRs aged ≥18 years, with at least a 10-year follow-up. A slower decline of eGFR over time was observed in active RTRs compared to non-active patients [148]. It is unclear how physical activity may protect one from kidney function deterioration. There is evidence that excessive weight, glucose intolerance, and high blood pressure are reduced by regular physical activities, indirectly avoiding eGFR decline [149]. In a recent paper, 19 RTRs prospectively underwent magnetic resonance images on a 3T scanner including diffusion-weighted, blood oxygenation level dependent (BOLD), and arterial spin labeling sequences in hip positions 0^0^ and >90^0^ before and after intravenous administration of 20 mg furosemide. Unexpectedly, graft perfusion values were significantly higher in flexed, compared to neutral hip position. BOLD-derived cortico-medullary R2 ratio was significantly modified during hip flexion, suggesting an intrarenal redistribution of the oxygenation in favor of the medulla and to the detriment of the cortex. Although more data are needed, the authors suggest avoiding prolonged sitting for RTRs and favor exercises without major sustained hip flexion, such as rowing, for example [150].

### 2.3. Diet

Nutrition is an important medical aspect in kidney transplantation. During the acute post-transplant phase, some patients with a long history of CKD and dialysis are generally malnourished [151,152]. In these patients, dietetic regimen of 25–35 kcal/kg ideal body weight (IBW)/day and 1.0–1.2 g protein/kg IBW/day is recommended, until achievement of nutritional adequacy [153,154].

In the long-term, a low-salt and high-fiber diet is recommended to prevent and treat obesity, diabetes, dyslipidemia, and hypertension. The Mediterranean diet exerts beneficial effects on CVD, diabetes, obesity, metabolic syndrome, and cancer [155,156,157,158,159,160]. The Mediterranean diet, including high consumption of olive oil, legumes, unrefined cereals, fruits, and vegetables, a moderate to high consumption of fish, a moderate consumption of dairy products (mostly as cheese and yogurt), a moderate wine consumption, and a low consumption of non-fish meat products, might also protect kidney graft function. In a study, the nine-point Mediterranean Diet Score was assessed in 632 RTRs with graft functioning for ≥1 year, adherent to the Mediterranean diet. During median follow-up of 5.4 years, the Mediterranean Diet Score was inversely associated with graft failure, kidney function decline, and graft loss, independently of potential confounders [161]. For people unfamiliar with the Mediterranean diet, this diet is based on a balanced diet of a variety of fresh fruits and vegetables, lean meats, and plenty of water, whereas fat dairy products and whole grains should be reduced.

### 2.4. Infections

Any severe infection can damage the kidney graft either because of rejection due to reduced immunosuppression or because of treatment with nephrotoxic anti-infective drugs such as aminoglycosides, vancomycin, amphoterin, quinolones, anti-fungal, and anti-viral agents. Urinary tract infections (UTI) are frequent but usually respond well to antimicrobials or do not need treatment if asymptomatic. However, frequent recurrence of UTI may be associated to an increased risk of chronic rejection [162]. UTI may induce acute pyelonephritis, particularly in patients with stones, stents, or mechanical obstruction. Rapid increase of serum creatinine is possible in pyelonephritis. Acute kidney damages may be reversible if infection is controlled, but may also cause graft loss and death [163] when bacteremia leads to multi-organ dysfunction, with the kidney frequently involved.

Candidosis, aspergillosis, and mucormycosis may cause severe renal disease usually in the early post-transplant period. BK polyoma virus is the most frequent cause of infective late graft failure. Infection occurs during childhood and remains latent in the tubules. In kidney transplant recipients, the reactivation of BK virus may occur. The diagnosis of BKV is made between 6 and 12 months post-transplantation, more often after treatment of rejection and in patients receiving strong immunosuppression [164,165]. BK virus infection may cause interstitial nephritis with a large number of plasma cells that leads to progressive graft loss. The prevalence of polyomavirus BK nephropathy in RTRs ranges between 3% and 8% [166,167,168]. The presence of viral inclusions, known as “decoy cells,” in urine and the presence of BK virus DNA in plasma and in urine are markers for the replication of BK virus infection [166,167]. The sensitivity of decoy cells is 100% but the predictive value is low, only 27%. The demonstration of viremia confirm the diagnosis, with a sensitivity of 100%, a specificity of 92% and a predictive value of 74% [169]. Renal biopsy shows interstitial nephritis with infiltrate rich in plasma cells, and atypical intra-nuclear viral inclusion bodies. Ureteral stenosis and/or hemorrhagic cystitis can also occur. In the presence of renal function, deterioration and positive PCR-BKV renal biopsy is mandatory. There is no specific treatment for BK virus infection. Reduction of immunosuppression may stabilize renal function in a few cases but may expose one to the risk of acute rejection. A systematic review showed that leflunomide, cidofovir, and intravenous immunoglobulins failed to show any efficacy [170]. Graft loss occurs in 15–50% of BK polyomavirus-associated nephropathy. The Transform study has recently shown that everolimus, in combination with CNI, has a protective effect on BKV infection development. If a patient has lost the transplanted kidney because of BKV nephropathy, in the absence of BKV viremia, retransplantation is not a contraindication [171], and the immunosuppressive regimen including everolimus may be protective from reinfection.

In conclusion, today many kidney transplant are lost because of death with functioning graft (47%), chronic allograft nephropathy (29%), acute rejection (2.8%), hyperacute rejection (0.1%), nonlethal cardiovascular events (2%), technical complications (0.8%), recurrent glomerulonephritis (3.2%), noncompliance (2%), and other causes (11.8%) [172].

A personalized approach to care for clinical signs leading to nonimmune kidney graft failure, including adherence to prescriptions, avoiding drug toxicities, controlling arterial hypertension, treating dyslipidaemia, caring for new onset diabetes mellitus, treating hyperuricemia, and suggesting a healthy lifestyle may contribute to prolonged renal transplant function, and give better and longer lives to transplant recipients.

## Data Availability

The study did not report any data.
